# A scoping review of interaction dynamics in minimally verbal autistic individuals

**DOI:** 10.3389/fpsyg.2024.1497800

**Published:** 2024-11-13

**Authors:** Olivia Boorom, Talia Liu

**Affiliations:** ^1^Speech-Language-Hearing: Sciences and Disorders, University of Kansas, Lawrence, KS, United States; ^2^Department of Speech, Language, and Hearing Sciences, Boston University, Boston, MA, United States

**Keywords:** coordination, synchrony, nonspeaking, autism spectrum disorder, contingency, social interaction

## Abstract

Interaction dynamics provide information about how social interactions unfold over time and have implications for communication development. Characterizing social interaction in autistic people who are minimally verbal (MV) has the potential to illuminate mechanisms of change in communication development and intervention. The purpose of this scoping review was to investigate the current evidence characterizing interaction dynamics in MV autistic individuals, methods used to measure interaction dynamics in this population, and opportunities for future research. Articles were included if participants were diagnosed with autism, considered MV, if interaction occurred with a human communication partner during live in-person interaction, and if variables were derived by measuring the relationship between behaviors in both partners. The seven articles included in this review demonstrate that limited research describes interaction dynamics in this population, and that behavioral coding measures can be leveraged to assess constructs such as turn-taking, social contingency, and balance in social interactions. While there is some evidence describing how MV autistic individuals and their communication partners construct reciprocal interaction, there is variability in how interaction dynamics are measured and limited evidence describing individual differences. Recommendations for future research are discussed.

## Introduction

Social interaction is a complex and dynamic process that serves as a vehicle for language development, learning, and relationship building (Feldman, 2007; Harrist and Waugh, 2002). Studying the nature of social interaction and how it unfolds (i.e., interaction dynamics) can provide valuable insight into successes or breakdowns in social communication, particularly in populations who display differences in social interaction, such as autistic people (American Psychiatric Association, 2013; Ruble et al., 2008; Sterponi et al., 2015). Beyond the characteristic differences in social communication related to an autism diagnosis (American Psychiatric Association, 2013), autistic individuals may display unique social interaction characteristics such as verbal routines, echolalia, and differences in temporal synchrony with a partner (Quigley et al., 2016; Sterponi and Shankey, 2014; Zadok et al., 2022).

### What are interaction dynamics?

The term interaction dynamics is used here as an umbrella term for multiple concepts related to how social partners influence each other and co-create meaning during social interaction (De Jaegher, 2013). Related concepts that fall under the umbrella of interaction dynamics include interpersonal synchrony, reciprocity, behavioral or interpersonal coordination, alignment, behavioral attunement, and social contingency, among others (see Provenzi et al., 2018 for review). Critically, interaction dynamics involve the contribution of two partners that adapt and respond to each other dynamically over the course of an interaction (Jaffe et al., 2001). In other words, describing the behavior of one partner (e.g., parental responsiveness) is not sufficient for measuring interaction dynamics. Rather, measuring the relationship between the actions of *both* partners to each other would more adequately describe their interaction dynamics.

Interaction dynamics can be measured using various methods, including automatic movement or physiological analysis, behavioral coding and annotation, conversation analysis, and global rating scales (Delaherche et al., 2012; Dumas and Fairhurst, 2021; Xu et al., 2020). In terms of qualitative ratings, live or video-recorded interactions of participants and a communication partner are typically rated by a third-party observer. For example, constructs such as synchrony, connectedness, and reciprocity can be measured by researchers using rating systems such as the Coding Interactive Behavior system (Feldman, 1998; Leclère et al., 2016), Infant Caregiver Engagement Phases (Cohn and Tronick, 1988) and the Joint Engagement Rating Inventory (Adamson et al., 2020). Observational behavior coding is another tool for assessing interaction dynamics. Turn-taking, for example, that has been examined extensively in the literature (see Nguyen et al., 2022 for review). Other methods include coding verbal and nonverbal behaviors of both communication partners and examining their contingency or coherence using cross-correlation, sequential analysis, recurrence analyses, or other methods (see Xu et al., 2020 and Dumas and Fairhurst, 2021 for review). Observational behavior coding often requires significant time and resources, though it provides micro-analytic information which can be used to quantitatively measure constructs such as coordination, synchrony and social contingency (Yoder et al., 2018; Yoder and Tapp, 2004). Qualitative ratings like those described above are less time- and resource-intensive than observational coding, however, they provide less detailed information about timing and contingency of individual behaviors. The process of coding behavioral data during dyadic interaction can also be automated, which yields results comparable to manually coded data, though automated coding may not be well suited to describe qualitative indicators of communication behavior such as directedness (Fujiwara et al., 2021). Conversation analysis blends both qualitative and micro-analytic approaches by analyzing actions and suprasegmental information (e.g., intonation, overlapping speech, and word stress) to describe the organization and structure of an interaction (Bottema-Beutel et al., 2021; Hepburn and Bolden, 2012). While behavioral coding techniques and analysis may provide valuable insight into the temporal structure of an interaction, conversation analytic techniques describe the social action structure.

### Contributions of interaction dynamics to communication development

Evidence shows that interaction dynamics such as social contingency, synchrony, and coordination play a key developmental role in infant and child language and social learning (Harrist and Waugh, 2002). From the first few months of life, infants and their caregivers coordinate their facial expressions and body movements and create contingent chains of behavior all before infants have developed intentional communication (Beebe et al., 2016; Fogel and Thelen, 1987; Kaye and Fogel, 1980). In fact, dyadic synchrony and attunement with caregivers promotes symbolic language development (Feldman, 2007; Feldman and Greenbaum, 1997), and greater dyadic synchrony at 12 months is associated with greater receptive and expressive language skills at 3 years (Kellerman et al., 2020). As infants develop, they play a more active role in social interactions and adapt their timing and turn-taking patterns to their caregivers (Abney et al., 2017; Cohn and Tronick, 1988; Hilbrink et al., 2015; Xu et al., 2020). Interaction dynamics continue to support language and social skills throughout the toddler years and early childhood (Harrist and Waugh, 2002; Lindsey et al., 2009).

### Importance of interaction dynamics in autism

Beyond the importance of reciprocity as a hallmark feature of autism, differences in social interaction may also have downstream effects on later developmental outcomes (Bradshaw et al., 2022). For example, children who are less engaged in reciprocal and synchronous communication with a partner (e.g., fewer bouts of engagement and communication) may have fewer opportunities for language and social learning. Evidence shows that parents and their children adapt to each other and attune their behaviors to each other, demonstrating the bidirectional effects of their individual behaviors on the interaction (Bottema-Beutel et al., 2018; Choi et al., 2020; Cohn and Tronick, 1988; Fusaroli et al., 2019). Therefore, examining social interaction dynamics rather than their individual elements may prove more powerful in capturing this complex relationship.

Social interactions, especially parent–child interactions, are integral to treatment approaches targeting autistic children’s language and communication skills. Treatment strategies for naturalistic developmental behavioral interventions for autistic children include routines-based activities, contingent responding, and imitation (Schreibman et al., 2015). Children participating in treatment approaches that include interpersonal synchrony as an active ingredient (as proposed in Green and Garg, 2018) have demonstrated increased social communication behaviors, such as imitation (Green et al., 2010; Green and Garg, 2018; Landa et al., 2011).

Taking a dynamic systems perspective that examines how interaction partners co-create a complex, changing system through their interaction, as opposed to examining individual partners’ communicative behaviors separately, enables the examination of social interaction theories in autism, such as the double empathy problem and the social motivation theory of autism (Milton, 2012; Thelen and Bates, 2003). The social motivation theory hypothesizes that differences in social interaction in autism are related to deficits in reward processing of social stimuli (*see* Bottini, 2018 *for review*). In contrast, the double empathy problem refers to a breakdown in interaction between people of different neurotypes and “personal conceptual understandings when attempts are made to communicate meaning” (Milton, 2012, p. 885). Importantly, the double empathy problem shifts understanding of social communication breakdowns in autistic-non-autistic dyads away from an individual-level problem (e.g., social reward processing) to an interpersonal problem in achieving mutual understanding (*see* Livingston et al., 2024 *for critique*; Milton et al., 2022; Milton, 2012). Measures of interaction dynamics are well-suited for examining social interaction from a double empathy problem lens, as interaction dynamics account for the contributions of *both* social partners.

### Interaction dynamics in minimally verbal and nonspeaking autistic individuals

Research with infants at elevated likelihood of autism (Kellerman et al., 2020; Northrup and Iverson, 2015; Steiner et al., 2018; Yirmiya et al., 2006) and with verbally fluent autistic children and adults (Feldman et al., 2014; Georgescu et al., 2020; Zampella et al., 2020) has found that dyads with one autistic partner demonstrate lower interactional synchrony and coordination than non-autistic dyads. In many of the examples highlighted above, autistic participants were engaged in conversational tasks that required relative verbal fluency. Given that social interaction typically utilizes multiple modalities of verbal and nonverbal behaviors, it is expected that interaction dynamics may shift when one partner is predominantly nonspeaking. Because an estimated one-third of autistic children are considered minimally verbal (MV, i.e., use few spontaneous spoken words by the time they reach kindergarten) and may experience persistent difficulties with language and communication, investigating how social interaction unfolds through interaction dynamics is an important and understudied area of research (Tager-Flusberg and Kasari, 2013).

A challenge in adequately describing interaction in MV individuals is identifying appropriate methods. Relying on measures such as vocal coordination or vocal temporal structure is inadequate to fully capture the social communication strategies employed by MV participants and their interaction partners. One solution is to employ a behavioral coding approach that measures all possible communicative behaviors such as touch, proximity, gesture, and gaze in order to measure partners’ coordination or contingency each other (e.g., Van Keer et al., 2019). Another approach is to assess qualitative features of interaction by rating levels of fluency, reciprocity, or contingency (e.g., Adamson et al., 2020). Additionally, some studies have used conversation analytic approaches to characterize the organization of interaction in speaking autistic children, which can be adapted for MV individuals (e.g., Bottema-Beutel et al., 2021). Given the importance of interaction dynamics in supporting communication development and evidence that language skills may be associated with individual differences in interaction dynamics, further investigation of interaction in individuals with limited language is warranted.

The literature related to interaction dynamics in autism is sparse and may span multiple fields and empirical approaches across psycholinguistics, developmental psychology, speech and language, and signal processing (Delaherche et al., 2012), therefore we determined that a scoping review was most appropriate to identify and synthesize as much available literature on this topic (Tricco et al., 2016, 2018). The purpose of this review is to (a) summarize the current evidence describing dyadic interaction dynamics in social interaction with MV autistic individuals, (b) describe methodologies used to measure interaction dynamics in this population, and (c) identify opportunities for future research.

## Methods

Following guidelines set by the PRISMA extension for scoping reviews, we first conducted a systematic search of articles. Articles were then screened by title, abstract, and full text as detailed below (Tricco et al., 2018). Independent reliability was gathered for title, abstract, and full text screening and data extraction.

### Search procedures

A systematic search procedure was conducted in August 2023 using ProQuest Research Library through the (masked for review) and PubMed. Articles gathered using ProQuest were extrapolated from 66 databases. The publication span searched was 1960–2023 and search terms were applied to the title and abstract. Search terms included the following: “autism,” “autistic,” “autisms,” “ASD” AND “minimally verbal,” “preverbal,” “pre-verbal,” “nonverbal,” “non-verbal,” “nonspeaking” AND “behavioral contingency,” “behavioral contingency,” “contiguity,” “turn-taking,” “mimicry,” “interactional dynamics,” “interaction dynamics,” “synchrony,” “reciprocity,” “attunement,” “coordination,” “social feedback,” “dyadic,” “bidirectional,” “responsiveness,” “alignment,” “coupling,” “mirroring.” The initial search yielded 494 articles. Duplicates were removed, resulting in a total of 259 unique articles. First, a title screening was conducted, then abstracts were screened, then remaining articles were screened for inclusion using the full text (screening procedures detailed below). Inter-observer reliability was calculated by dividing the number of agreements by the total number of agreements and disagreements (Tricco et al., 2018).

#### Inclusion and exclusion criteria

Peer-reviewed articles, theses, dissertations, and preprints were screened to meet the following inclusion criteria: (a) participants were diagnosed with autism, (b) participants, or a subgroup of participants, were identified as being MV or having documented verbal language abilities consistent with early word production, (c) interactive behavior was measured from a face-to-face interaction with another human communication partner, (d) the relationship between interaction behaviors in both partners were measured (e.g., contingency between caregiver communication and child communication) or interactive variables by definition included behavior from both partners (e.g., synchrony). Exclusion criteria were as follows: (a) articles were not available in English, (b) participants had other genetic and/or neurodevelopmental disorders (e.g., Down syndrome, fragile X syndrome, dup15q syndrome) (c) participants were described as verbally fluent, “high-functioning,” or Asperger’s syndrome, (d) participants included infants (< 18 months old), making limited spoken language developmentally appropriate, and (e) interactive behavior was measured with a robot, screen, or virtual human. A detailed screening and review protocol can be found in the Supplementary materials.

#### Title screening

The titles of all 259 articles were screened by the first author (primary coder) and a graduate student research assistant (reliability coder) for mention of (a) autism and (b) social interaction or any face-to-face interactions including interventions, parent–child play and/or natural observation. Titles were excluded if they indicated that participants were diagnosed with a non-autism genetic and/or neurodevelopmental disorder, participants were infants, participants were “high-functioning” or diagnosed with Asperger’s syndrome, and if the article was an opinion, commentary, or review without original data. Reliability for the title screening was 86%. Titles that were included by only one coder were also included in the abstract screening. The title search yielded 101 articles.

#### Abstract screening

Next, the abstracts of the 101 articles that passed the title screening were screened using the following additional inclusion criteria: (a) participants were characterized as being MV, nonverbal, or language in the early word production stage, (b) study procedures included dyadic interactions, as opposed to group interactions, and (c) interaction variables were measured as a relationship between behaviors in more than one partner. Reliability for the abstract screening was 83%. If the abstract included vague or unclear information about any of the above inclusion criteria, they were included for full text screening. Abstracts that were marked for inclusion by only one author were included in the full text screening. The abstract screening yielded 43 articles for further review.

#### Full text screening

Articles that passed the abstract screening were read in full and reviewed for final inclusion/exclusion. Specifically, participant characteristics were analyzed to assess whether participants met criteria for MV status. In the case of single case experimental designs, studies were included if at least one participant was characterized as MV. Additionally, methods were reviewed to determine whether interaction variables met our definition of interaction dynamics. Namely, variables must measure the relationship between behavior in both members of the dyad. Agreement for the full text screening was 84.2%, and all disagreements were reviewed for consensus until agreement reached 100%.

### Data extraction and synthesis

Once all articles were screened and reviewed for final inclusion, the following data were extracted by the first and second author: participant ages, diagnoses and language skills, operational definitions of “minimally verbal” or nonspeaking, definitions of interactional variables, coding measures or other measurement methods, and results related to interactional variables. The data extraction template (adapted from Dada et al., 2021) can be found in the Supplementary materials. The percentage agreement for data extraction was 95.2% and all disagreements were discussed to reach consensus. Data were synthesized with the goal of identifying common methods across studies to measure interaction and identifying similarities and differences in observations and descriptions of interactional variables.

## Results

**Figure 1:**
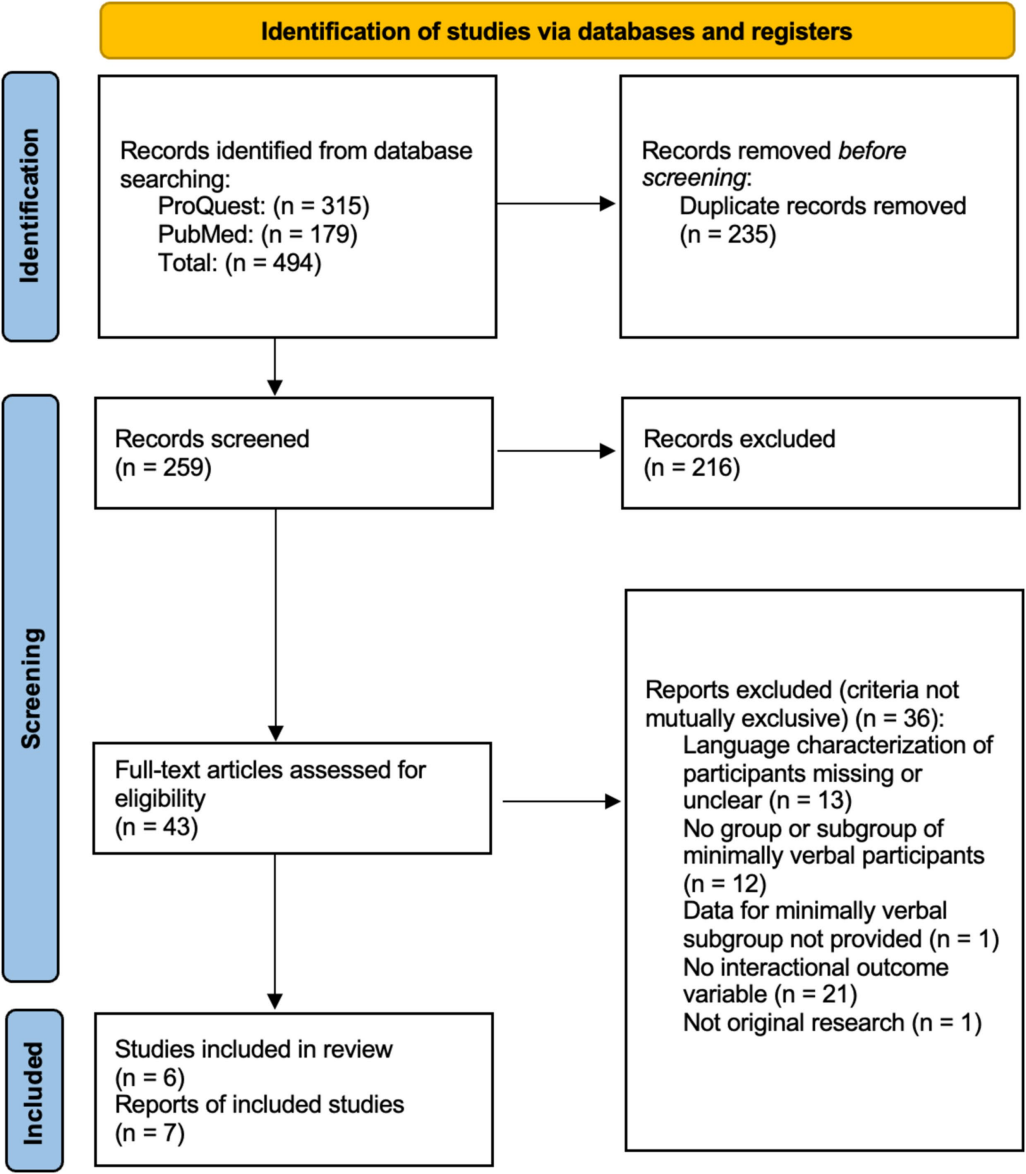
PRISMA flow diagram.

The literature search yielded seven eligible papers, including one cross-sectional observational study (Doussard-Roosevelt et al., 2003), one randomized controlled trial (Thiemann-Bourque et al., 2018), two single case experimental designs (Bourque and Goldstein, 2020; Ishizuka and Yamamoto, 2016), and three single observation case studies (Chen, 2022; Delafield-Butt et al., 2020; Lee et al., 2023). The full search process and strategy is outlined in the PRISMA flowchart (Page et al., 2021) in Figure 1.

### Participant demographics

Of the seven articles reviewed here, 63 participants were considered MV. Two articles drew from the same cohort of participants (Bourque and Goldstein, 2020; Thiemann-Bourque et al., 2018). Participant ages ranged from 2 years and 4 months to 18 years. The majority of participants were preschoolers, and all participants with the exception of one 18-year-old woman were under 6 years old. Communication partners featured were an interventionist in two studies, a parent in three studies, and a neurotypical peer in two studies (from the same cohort). All seven reports measured interaction between an autistic participant and a non-autistic communication partner.

#### Definition of minimally verbal/nonspeaking

In line with reviews of literature pertaining to MV and nonspeaking individuals, studies varied greatly in their definitions of MV and the assessment procedures used to characterize participants (Koegel et al., 2020). Five of the seven reports explicitly described participants as MV or nonverbal, though inclusion criteria for characterization as MV differed among studies. Two case studies described participants as nonverbal or non-speaking, though specific spoken language characterization was not provided (Chen, 2022; Delafield-Butt et al., 2020). Doussard-Roosevelt et al. (2003) distinguished between verbal and nonverbal children based on maternal report of either presence or absence of words, whereas Thiemann-Bourque et al. (2018) characterized children as MV if they used fewer than 20 spontaneous words. One single case design study assessed participants with varied language skills, therefore participants met criteria for MV status; only this subset’s characterization of interaction dynamics is reported here (Ishizuka and Yamamoto, 2016). Though participants were not specifically characterized by the authors as MV, narrative descriptions of participant baseline language skills indicated that they had few to no spontaneous words (Ishizuka and Yamamoto, 2016).

### Measures of interaction dynamics

Variables used to measure interaction dynamics included turn-taking, balanced initiations and responses, engagement, and social contingency. Operational definitions of interaction variables are provided in Table 1. Importantly, many of the studies here measured the larger construct of reciprocity or reciprocal interaction. All studies involved some degree of manual behavior coding, though Thiemann-Bourque et al. (2018) and Bourque and Goldstein (2020) coded interaction behaviors *in vivo* while all other studies used data coded offline via video recording.

**Table 1:** Eligible studies and definitions of variables measured.

Article	Sample size (MV participants)	Participant ages	Interaction variable	Definition
Bourque and Goldstein (2020)	6 (males = 2)	3 years 7 months - 5 years 1 month	Balanced initiations and responses	Proportion of responses (vs. initiations) out of communicative acts. “Initiations were coded based on who started communicating first and/or if a minimum of 3 s had passed since the last communication act (by either the focus child or the peer).
				Responses were coded if the other communication partner responded to the previous initiation within 3 s or in response to a partner’s previous response within 3 s.”
Chen (2022)	1 (males = 1)	5 years	Multi-turn constructions	“a collaborative verbal sequence between interlocutors that takes place over multiple turns, and that is repeated across interactional contexts.”
Delafield-Butt et al. (2020)	1 (males = 0)	18 years	Engagement periods	“Periods of engagement between the therapist and patient during which time expressive action was either (a) attempted by one or the other person or (b) expressed by one person and responded to by the partner, indicating that it had been treated as if it were communicative even if it was unlikely that the partner had intended it in this fashion, or (c) an act was delivered and received as communicative”
Doussard-Roosevelt et al. (2003)	12 (gender information not provided)	3 years - 5 years 9 months	Contingency	A contingency profile was calculated using each combination of maternal approach behavior and child response type. “Percentage scores…reflected the overall level of child contingency to the mother’s Approach behaviors…”
Ishizuka and Yamamoto (2016)	3 (males = 3)	2 years 9 months - 5 years 3 months	Vocal turn-taking	“A behavior chain in which child and adult vocal responses occurred continuously in each. We counted “child speech and vocalization to adult speech and vocalization” or “adult speech and vocalization to child speech and vocalization” as one turn each. From 3 s when the last adult speech and vocalization occurred, we recounted as another unit of vocal turn-taking from the next speech and vocalization.”
Lee et al. (2023)	1 (males = 1)	2 years 4 months	Social turn-taking	“…playful, back-and forth exchanges for the purpose of engaging socially with a communicative partner.”
				Instrumental turn-taking	“back-and-forth exchanges for the purpose of following or initiating requests.”
			Thiemann-Bourque et al., 2018	45 (males = 36)	2 years 11 months - 5 years	Balanced initiations and responses	Proportion of responses (vs. initiations) out of communicative acts. “Initiations were coded based on who started communicating first and/or if a minimum of 3 s had passed since the last communication act (by either the focus child or the peer).
Responses were coded if the other communication partner responded to the previous initiation within 3 s or in response to a partner’s previous response within 3 s.”						

#### Turn-taking

Three studies included in this review measured vocal or nonverbal turn-taking (Chen, 2022; Ishizuka and Yamamoto, 2016; Lee et al., 2023). Definitions of turn-taking varied slightly. Ishizuka and Yamamoto (2016) defined turn-taking as any adult-child or child-adult turn counts (i.e., two-event turns) that occurred within a 3 s latency. In contrast, Chen (2022) defined multi-turn constructions as collaborative verbal sequences that include at least two turns (i.e., three-event turns). As others have noted, two-event bouts may be more closely related to measures of partner or child responsiveness, rather than measures of reciprocal interaction, while three-event (or higher) bouts indicate a continuing interaction between partners (Harbison et al., 2018). Operational definitions of turn-taking also differed in their inter-turn interval requirements.

#### Balanced initiations vs. responses

In a peer-mediated AAC intervention for MV autistic preschoolers, Bourque and Goldstein (2020) and Thiemann-Bourque et al. (2018) assessed the proportion of communicative responses and communicative initiations. Participants who engaged in more well-balanced reciprocal interaction exhibited a greater proportion of responses to initiations, suggesting that interactions continued for extended periods.

#### Engagement

Delafield-Butt et al. (2020) measured the frequency, complexity, modality, and amount of time spent in periods of engagement between a participant and interventionist. Engagement periods were defined as periods where an “expressive action” (i.e., an action that a partner could interpret as communicative) was attempted by either partner, regardless of whether it was responded to.

#### Social contingency

One study used social contingency as a measure of interaction by identifying the proportion of maternal approach behaviors that elicited a child response in a contingency profile derived from the Approach-Withdrawal Interaction Coding System (Doussard-Roosevelt et al., 1995, 2003). Approach behaviors were defined as social, physical, or object-focused attempts to engage the child with a minimum of 3 s pause to allow the child to respond. The authors classified child responses as either approaches or withdrawals based on whether children attempted to continue the interaction (approach) or not (withdrawal) (Doussard-Roosevelt et al., 2003).

### Characteristics of interaction in MV autistic individuals

All seven studies described MV autistic participants as engaging in some degree of reciprocal interaction, though the actions that participants used to engage with a partner may be different than expected for their age. For example, Delafield-Butt et al. (2020) found that the young woman and interventionist demonstrated over a dozen engagement periods that increased in length and complexity over the course of the interaction. Rather than words or conventional gestures (which would be expected communication modalities for a young adult), the participant often used expressive actions such as stomping her foot, slapping the table, or changes in proximity that were then scaffolded by the responsive interventionist (Delafield-Butt et al., 2020). The authors measured the narrative phases (initiation, build, climax, and conclusion) and the number of acts included in each engagement period and found that they both increased over the course of the total interaction, indicating increased engagement and reciprocity from the participant. Similarly, Chen (2022) found that a young autistic participant and his mother exhibited many vocal multi-turn constructions that were highly routinized, repeated across contexts and activities, and unfolded at a rapid tempo, even while the participant primarily used non-word vocalizations.

Reciprocal interaction behaviors such as turn-taking also occurred at a reduced rate compared to developmental expectations. Lee et al. (2023) measured social and instrumental turn-taking behaviors in a pilot study of a parent-mediated social communication intervention. The toddler in that case study demonstrated social turn-taking behaviors in only an average of two out of 60 10-s intervals at baseline (Lee et al., 2023). Similarly, Doussard-Roosevelt et al. (2003) found that autistic preschoolers demonstrated less social contingency to their mothers’ approach behaviors than non-autistic preschoolers. However, when they examined group differences in social contingency based on language ability, they found no differences in the proportion of child contingency to mothers’ approaches between nonverbal and verbal autistic children, regardless of the mothers’ type of approach behavior (Doussard-Roosevelt et al., 2003). Thiemann-Bourque et al. (2018) found that at baseline, MV autistic preschoolers demonstrated unbalanced communicative interactions with many more initiations than responses. Peers also showed few responses compared to initiations, suggesting that interactions between participants and peers were short and/or communicative bids were frequently dropped by either partner. Low rates of communication between partners was consistent across multiple communicative functions, including requests for objects, actions, and joint attention as well as comments (Bourque and Goldstein, 2020). Overall, studies consistently showed that across communication partners and contexts, nonspeaking autistic individuals used a variety of communicative acts to engage in reciprocal interaction and did so less frequently than expected for their age.

### Factors related to differences in interaction dynamics

Some of the studies in this review also examined various predictors of interaction dynamics. While not assessed systematically, Delafield-Butt et al. (2020) noted that in the observed periods of engagement between a young autistic woman and an interventionist, the interventionist frequently used imitation to respond to the woman’s expressive actions. Consistent with this observation, Ishizuka and Yamamoto (2016) found that all three MV children in their social communication intervention demonstrated higher rates of vocal turn-taking when an interventionist used contingent imitation compared to general contingent responding.

Multiple intervention trials in this review saw increases in interactive behaviors in response to intervention. In a parent-mediated intervention targeting social communication skills through increased parent–child engagement and turn-taking activities, Lee et al. (2023) found that turn-taking increased during intervention but was not maintained post-intervention. Thiemann-Bourque et al. (2018) found that peer-mediated intervention using a Stay, Play, Talk approach to teach responsive play and communication led to more balanced proportions of initiations and responses in MV preschoolers.

## Discussion

The aims of this scoping review were to summarize the current evidence describing dyadic interaction dynamics in social interaction with MV autistic individuals, describe methodologies used to measure interaction dynamics in this population, and identify opportunities for future research.

This review identified only seven studies examining interaction dynamics in MV autistic individuals. The lack of studies in this area is notable considering current evidence that interaction dynamics differ in autistic children (Feldman et al., 2014; Georgescu et al., 2020; Zampella et al., 2020) and the significant heterogeneity of communication outcomes in this population. The majority of studies identified in this review (five of seven) were either single case experimental designs or case studies. Though these studies offered rich and detailed characterization of individual participant interactions, broader generalizations about the larger population of MV autistic individuals cannot be drawn from these data. However, these case studies provide important methodological implications for future research. Methods used to measure coordination or synchrony in typically developing infants may require adaptation to meaningfully capture the nature of social interaction in individuals with limited speech. For example, one participant engaged with her therapist using many behaviors with ambiguous communicative intent, such as stomping her feet or moving her body toward the therapist (Delafield-Butt et al., 2020). The descriptions of participant behaviors provided in that case study, alongside the low rates of vocal behavior described in Ishizuka and Yamamoto (2016), highlight the need to consider a broad range of behaviors beyond vocalizations, gaze, and gestures when measuring interaction in this population.

Overall, the studies reviewed here demonstrate that MV autistic individuals engage in some degree of reciprocal interaction with their communication partners and that these interaction dynamics can be successfully analyzed using a variety of outcome measures. It is unclear from the current evidence whether interaction dynamics in MV individuals differ from those in verbal autistic children. Only one study compared interaction dynamics between verbal and MV autistic children and found that MV participants’ social contingency to maternal behavior differed from typically developing children but not from verbal autistic children (Doussard-Roosevelt et al., 2003). Future examination of other aspects of interaction, such as temporal coordination or length of turn-taking bouts, may reveal between-group differences in how social interaction unfolds.

### Measures of interaction dynamics

With the exception of Doussard-Roosevelt et al. (2003), there was a notable absence of quantitative methods for measuring interaction dynamics beyond frequency counts of specific behaviors in the literature in this review. One potential explanation is that many previous studies of interactional synchrony in typically developing children have relied on vocal/verbal behavioral synchrony, which occurs at a reduced rate in MV children (Abney et al., 2017; Fujiwara et al., 2021). While their application may be less suited to intervention studies like many of those reviewed here, quantitative analysis of interaction dynamics offers the ability to make inferences about the *degree* of reciprocity. Such measures can be standardized to compare among participants. Further, simulated data sets can be created as controls to compare the degree of reciprocity against chance-level associations (Delaherche et al., 2012; Lourenço et al., 2021).

All seven studies in this review relied on manual behavior coding or transcription of interactions either *in vivo* or offline. While all variables of interest measured the relationship between participant and partner behavior during social interactions, measures included turn-taking, balanced initiations and responses, engagement, and social contingency. Even among studies measuring similar interactional variables, operational definitions varied. Turn-taking measures, for example, were inconsistent in their latency parameters between turns, minimum number of turns, and behaviors of interest (Chen, 2022; Ishizuka and Yamamoto, 2016; Lee et al., 2023). Challenges with defining optimal parameters for turn-taking specifically have been previously described in the literature, particularly due to the lack of consensus on inter-turn latencies in interactions with autistic children (Bottema-Beutel and Kim, 2021). Only one study utilized a previously established coding scheme to measure social contingency (Doussard-Roosevelt et al., 1995); all other reports used coding schemes that were either untitled or designed for the study, making replication and generalization across reports challenging (Uzonyi et al., 2023). Use of validated coding systems and methods for measuring interaction dynamics in future studies in this population would enable replication, interpretation of results, and consistency with terminology.

### Research directions

Based on the gaps in the literature identified in the current review, there are several areas for future research:

1. Validated quantitative measurement of interaction dynamics. As described above, most articles relied on frequency counts of interactive behaviors to quantify dynamics in dyadic social interactions. While frequency counts and rates can help establish base rates of behavior or track improvement over time, (e.g., response to intervention; Lee et al., 2023), they fail to describe how dynamics *unfold* over the course of an interaction or describe which partner is exerting more influence over the flow of the interaction. The absence of synchrony variables, for example, is notable considering that dyadic synchrony is a potential mechanism and mediator of many autism interventions (Green and Garg, 2018; Landa et al., 2011). Future research can expand this work by adapting measures commonly used in the parent-infant interaction literature for use with MV autistic individuals across the lifespan and assess their social validity through third-party clinical judgment (Abney et al., 2017; Northrup and Iverson, 2020; Xu et al., 2020). Even in the infant communication literature, researchers have highlighted that considering multiple modalities of communication is necessary for an accurate description of parent–child interactions (Kosie and Lew-Williams, 2024). Other promising advancements in signal processing and machine learning that future work can leverage the use of motion energy analysis, automated coding, and simulated data to assess at-chance levels of interaction variables and validate automated procedures (Georgescu et al., 2019; Harbison et al., 2018)
2. Interaction in autistic-autistic dyads. All seven studies measured interaction of autistic individuals with a neurotypical partner. Given the emerging evidence that interactions may be judged as smoother and more in-sync in same-neurotype dyads than in mixed-neurotype dyads, future research may examine how interaction dynamics unfold in autistic dyads where one partner is MV (Crompton et al., 2020; Georgescu et al., 2020). Research in this understudied area may help disentangle the contribution of autism traits and language skills in shaping dyadic interactions. Additionally, research in autistic-autistic interactions would allow for closer examination of behavioral evidence for theories of social interaction in autism, such as social motivation theory or the interactional heterogeneity hypothesis (e.g., double empathy problem). Specifically, rigorous qualitative and quantitative accounts of autistic-autistic interactions compared to mixed neurotype interactions would provide a more detailed account of how communication breakdowns and repairs, shared timing, and meaning making unfold in relation to differences in neurotype.
3. Predictors of individual differences in interaction dynamics. The qualitative studies described discuss partner behaviors that may facilitate increased engagement, turn-taking, and contingency between interaction partners (Chen, 2022; Delafield-Butt et al., 2020). Additionally, the three intervention studies share some treatment components common to many early interventions that may have promoted increased reciprocity, such as partner responsivity, wait time, and environmental arrangement, though these components’ contributions to increasing reciprocity in interaction were not assessed (Ishizuka and Yamamoto, 2016; Lee et al., 2023; Thiemann-Bourque et al., 2018). Considering that two studies described how contingent imitation was related to changes in engagement and turn-taking (Delafield-Butt et al., 2020; Ishizuka and Yamamoto, 2016), the impact of imitation on promoting reciprocal interaction warrants further examination. Because interaction dynamics continually change over the course of an interaction, understanding the specific antecedents and contexts that facilitate such changes can inform communication partner training and intervention planning.

### Limitations and conclusions

While outside of the intended scope of the present review, we did not evaluate the quality or levels of evidence of studies included in this review. As the literature in this area grows, future systematic reviews would help synthesize results across studies and assess the quality of current evidence. This is particularly relevant considering the limited sample sizes in many of the studies reviewed here. Another limitation of this review is potential language bias, as articles that were not written or translated in English were excluded. Finally, it is possible that some studies were missed during the initial search, particularly considering the varying terminology and criteria used to describe MV individuals (Koegel et al., 2020). Some studies were excluded in full-text screening because participants’ language characterizations were inadequate for determining their MV status. It is possible that some articles that indeed examined interaction in MV autistic individuals but did not use this terminology were excluded from the initial search.

In summary, this scoping review maps the available evidence on interaction dynamics in MV autistic individuals. This review demonstrates that while limited existing evidence has worked to describe interaction dynamics in this population, it highlights the need to further characterize social interaction using methods that consider the multimodal nature of communication and examine factors that influence interaction dynamics across partners, contexts, and individual behaviors.
