# Supplementary material for: A scoping review of interaction dynamics in minimally verbal autistic individuals
Source: Front Psychol. 2024 Nov 13;15:1497800. doi: 10.3389/fpsyg.2024.1497800 (PMC11598442; doi:10.3389/fpsyg.2024.1497800)
Supplement: Supplementary file 2 [file Data_Sheet_2.DOCX]

**Scoping Review Literature Search**

The purpose of this review is to find current literature describing interaction dynamics in minimally verbal and/or nonspeaking autistic children. Interaction dynamics refers to how to partners work together to create interaction, rather than behavior of a single partner. As a result, interactive variables account for BOTH partners – examples include coordination, synchrony, reciprocity, attunement, etc.

1. **Title Screening**

Use the title and the title only to guide whether you include/exclude papers at this step. The goal is to get rid of papers which have very little to do with the topic. In column F in your spreadsheet, type either “Y” or “N” to denote whether it’s included/excluded. It can also be helpful to color-code the whole row green vs red, for example.

EXCLUDE papers which:

- Specifically mention non-autism disorders such as Down syndrome, fragile X, genetic disorders, etc.
- Specifically mention “high-functioning” or Asperger’s syndrome.
- Mention robot interaction or virtual interaction
- Are not available in English (primary or translated)
- Are non-research articles – key words to look for include “systematic review”, “response to:”, “commentary”, etc.
- Are unnecessarily broad – e.g. “Diagnosis of autism”.

INCLUDE papers which:

- Specifically mention autism or ASD
- Mention face to face or interpersonal interaction, communication, nonverbal behavior
  - This DOES also include in-person intervention studies

When in doubt, include the paper so that it can go under more thorough review in the abstract screening phase.

1. **Abstract Screening**

Now use the abstract to guide whether you include/exclude papers at this step. To do this, either enter the DOI or the title of the paper into google scholar and scan the abstract (it’s ok if you don’t have access to the entire paper, online versions should always show you the abstract as a preview) to answer the questions below. Use the criteria below as a decision tree/checklist, moving through all of the options for each abstract. For example, to reach an answer of YES for question 1, the abstract must satisfy *at least* one criterium listed. Fill in your answers in the tracking spreadsheet: enter Yes, No, or Unclear.

1. Do participants include a group of minimally verbal autistic individuals?

Clear YES if…

- - - Description of the participants includes the terms “minimally verbal”, nonspeaking, preverbal, low verbal, no fluent speech, “early stages of language development”, or nonverbal.
    - AAC or speech-generating device intervention is the primary focus
    - Clear description of participant’s language level
    - Mention of any nonverbal subgroup

Clear NO if…

- - - Description of participants includes Asperger’s, high functioning autism, typical language/typical cognition
    - Participants are described as infants or infant siblings of children with autism
    - Tasks include verbal conversation (also look for key words like syntax and discourse)
    - The paper is not a research paper at all – a conceptual review, systematic review, opinion piece

UNCLEAR if…

- - - No obvious participant description – i.e., participants are at least toddlers or older, have autism, but no clear characterization of their language abilities

1. Are dyadic interactions isolated?

Clear YES if…

- - - Abstract mentions measuring parent-child interactions or peer-to-peer interactions
    - Abstract mentions parent-mediated intervention or any therapeutic intervention

Clear NO if…

- - - Study ONLY uses standardized measures or surveys of social interaction/social communication
    - The methods mention eye-tracking without other clear face to face interaction variables
    - Abstract mentions large group interactions only
    - Any neuro-imaging methods EXCEPT fNIRs

UNCLEAR if…

- - - Intervention study without clear description of outcome measures

1. Does the study measure interaction variables in more than 1 partner?

Clear YES if…

- - - Methods mention contingency between behaviors
    - Methods mention dyadic variables, i.e., synchrony, interpersonal coordination, attunement, mirroring, reciprocity
    - Methods describe measuring BOTH child and other partner behavior and relate them to each other in some way.

Clear NO if…

- - - Partner behavior is not mentioned
    - Parent responsiveness is the primary variable of interest without mention of child communicative behavior
    - Criterium #2 is not met

UNCLEAR if…

- - - Mention of parent-child, therapist-child, or peer-peer interaction without explanation of dependent variables/measures
    - Intervention study without clear description of outcome measures

If the answer to any of the questions above is NO, then the abstract does not pass. If all answers are either UNCLEAR or YES, then the abstract passes the screener.

1. **Full Text Screening**
2. Do participants include a group of minimally verbal autistic individuals?

Clear YES if…

- - - < 2.5 SD below mean of a language assessment (if available) or age equivalents </= 18m

AND < 20 spontaneous unique words in vocabulary

OR “very little spontaneous speech”

- - - described as “minimally verbal” “nonverbal” or “preverbal”
    - A group or single cases of minimally verbal participants are analyzed separately

Clear NO if…

- - - Description of participants includes Asperger’s, high functioning autism, typical language/typical cognition
    - Tasks include verbal conversation (also look for key words like syntax and discourse)
    - If the above “Yes” criteria are not met

1. Are dyadic interactions isolated?

Clear YES if…

- - - Methods include measuring parent-child interactions or peer-to-peer interactions
    - Parent-mediated intervention or any therapeutic intervention with a therapist/examiner

Clear NO if…

- - - Study ONLY uses standardized measures or surveys of social interaction/social communication
    - The methods mention eye-tracking without other clear face to face interaction variables
    - Group interaction
    - Any neuro-imaging methods EXCEPT fNIRs

1. Does the study measure interaction variables in more than 1 partner?

Clear YES if…

- - - Methods mention contingency between behaviors – e.g. correlation or contingency coefficient between partner and child behaviors for each dyad
      - Group-level correlations between parent and child behavior do not count, only a YES if they are on the individual dyad level.
    - Methods mention dyadic variables, i.e., synchrony, interpersonal coordination, attunement, mirroring, reciprocity
    - Methods describe measuring BOTH child and other partner behavior and relate them to each other in some way.

Clear NO if…

- - - Partner behavior is not mentioned
    - Parent responsiveness is the primary variable of interest without mention of child communicative behavior – or if parent and child behaviors are measured separately without any analysis of their relationship

If the answer to any of the questions above is NO, then the abstract does not pass. If all answers are YES, then the abstract passes the screener.
